# Racial disparities in nephrectomy and mortality among patients with renal cell carcinoma: Findings from SEER

**DOI:** 10.1371/journal.pgph.0001314

**Published:** 2023-05-23

**Authors:** Joshua Ikuemonisan, Taiwo Opeyemi Aremu, Isaac Oyejinmi, Christopher Ajala, Nnabuchi Anikpezie, Oyindamola Akinso, Mutsa Mtengwa, Adeyemo David, Olugbenga Olokede, Oluwakayode Adejoro

**Affiliations:** 1 Division of Tobacco Research and Prevention, Masonic Cancer Center, University of Minnesota, Minneapolis, Minnesota, United States of America; 2 Department of Pharmaceutical Care & Health Systems (PCHS), University of Minnesota, Minneapolis, Minnesota, United States of America; 3 Division of Environmental Health Sciences, School of Public Health, University of Minnesota, Minneapolis, Minnesota, United States of America; 4 Masonic Children’s Hospital, University of Minnesota, Minneapolis, Minnesota, United States of America; 5 Hospital Data and Analytics, HealthPartners, St Louis Park, Minnesota, United States of America; 6 Department of Radiology, Medical College of Wisconsin, Milwaukee, Wisconsin, United States of America; 7 Department of Population Health Science, The University of Mississippi Medical Center, Jackson, Mississippi, United States of America; 8 Department of Public Health, Nova SouthEastern University, Fort Lauderdale, Florida, United States of America; 9 Department of women’s Health, Greater Baltimore Medical Center, Baltimore, Maryland, United States of America; 10 Department of Nursing, Indiana Wesleyan University, Marion, Indiana, United States of America; 11 F. Maie Hall Institute for Rural and Community Health, Texas Tech University Health Sciences Center, Lubbock, Texas, United States of America; 12 Market Access, Janssen Global Services, LLC, Horsham, Philadelphia, United States of America; PLOS: Public Library of Science, UNITED STATES

## Abstract

**Purpose:**

To assess racial differences in the receipt of nephrectomy in patients diagnosed RCC in the US.

**Materials and methods:**

2005 to 2015 data from the SEER database was analyzed and 70,059 patients with RCC were identified. We compared demographic and tumor characteristics between black patients and white patients. We applied logistic regression to assess the association between race and the odds of the receipt of nephrectomy. We also applied Cox proportional hazards model to assess the impact of race on cancer-specific mortality (CSM) and all-cause mortality (ACM) in patients diagnosed with RCC in the US.

**Results:**

Black patients had 18% lower odds of receiving nephrectomy compared to white patients (p < 0.0001). The odds of the receipt of nephrectomy also reduced with age at diagnosis. In addition, patients with T3 stage had the greatest odds of receiving nephrectomy when compared to T1 (p < 0.0001). There was no difference in the risk of cancer-specific mortality between black patients and white patients; black patients had 27% greater odds of all-cause mortality than white patients (p < 0.0001). Patients who did not receive nephrectomy had a 42% and 35% higher risk of CSM and ACM respectively, when compared to patients who received nephrectomy.

**Conclusions:**

Black patients diagnosed with RCC in the US have a greater ACM risk and are less likely than white patients to receive nephrectomy. Systemic changes are needed to eliminate racial disparity in the treatment and outcomes of RCC in the US.

## Introduction

The main types of kidney cancer are renal cell cancer (RCC), transitional cell cancer, and Wilms tumor [1], with RCC being the most common type of kidney cancer and constituting approximately 90% of cancers derived from the renal parenchyma [2–5]. Kidney cancer is the 8th most common cancer in the United States (US) [6, 7]. This is a disease of public health importance; in the United States, the prevalence of kidney and renal pelvis cancer was 582,727 as of 2018. In 2021 alone, the estimated number of new kidney cancer cases in the US was 76,080, accounting for about 4.0% of all new cancer cases in the US in 2021 [7]. Kidney cancer is one of the top ten cancers that lead to death, accounting for 2.3% of all estimated cancer deaths in the US in 2021 [7].

Although kidney cancer impacts persons of all races, studies have shown that it does not always affect the races equally [8]. Literature is replete with evidence of racial disparities in health conditions and outcomes between black patients and white patients in the US and how the experiences of different races have shaped health outcomes [9]. Additionally, the incidence rates of kidney cancers have been rising more rapidly among black patients than white patients over the past five decades [10]. An analysis of the incidence and survival among patients with kidney cancer from the national Surveillance, Epidemiology, and End Results (SEER) Registry Database, showed that black patients have a more significant rise in incidence and a poorer outcome than white patients of the same age and disease stage [11].

Nephrectomy is the recommended surgical treatment for non-metastatic kidney cancers [12]. Due to discriminatory policies and practices, black patients are more likely to face barriers in accessing healthcare leading to worse treatment outcomes than their white counterparts. In addition, black patients have more renal-relevant comorbidities (hypertension, diabetes, and chronic renal failure) and poorer overall cancer survival compared to white patients [13, 14].

Studies evaluating disparity in nephrectomy by race have been few and many have yielded equivocal results, necessitating the investigation of the role of race (as a proxy for exposure to structural racism) [15] in the receipt of nephrectomy using a large and nationally representative database. Therefore, this study examines the presentation, early definitive surgical treatment, and mortality among patients with RCC in the US, to investigate racial disparity in the receipt of nephrectomy for renal cell carcinoma patients.

## Methods

### Data source

We analyzed SEER data of patients diagnosed with renal cell carcinoma between 2005 and 2015 in the United States. The SEER database is a product of a nationally representative surveillance conducted by the National Cancer Institute (NCI), which collects demographic, pathologic, and clinical information on incident cancer across 18 geographic locations in the United States, covering nearly a third of the US population. Our study cohort includes patients diagnosed with renal cell carcinoma between January 1, 2005, and December 31, 2015 [16].

### Study population

We identified 75,714 patients diagnosed with histologically confirmed non-metastatic renal cell carcinoma diagnosed between 2005 and 2015. We excluded patients who were diagnosed for the first time in nursing/ convalescent homes (n = 242), patients whose race were neither Black nor White (n = 5,029). Patients diagnosed in Louisiana in 2005 (n = 384) were also excluded. The records from patients diagnosed with RCC in Louisiana were excluded because of the possibility of a differential loss of records due to the impact of Hurricane Katrina in 2005. The final cohort consisted of 70,059 patients with renal cell carcinoma. The outcomes of the study were reviewed until December 31, 2015, or the patient’s demise.

### Ethical consideration

This study analyzed publicly available data from the SEER database, hence has been approved based on the broad agreement signed in the SEER data-use agreement. This agreement ensures the study satisfies the Health Insurance Portability and Accountability Act of 1996 (HIPAA), confidentiality, and ethical considerations.

### Variables

The baseline demographic data include age (10–year intervals), race was categorized as “Black” vs “White” irrespective of ethnicity, tumor stage (T1, T2, T3, and T4), receipt of nephrectomy (Yes vs No), binary survival status (Dead (Coded as “0”) vs Alive (Coded as “1”)), and SEER registry geographic location according to the Census Bureau of the United States (Northeast, Midwest, South, and West). We defined survival as the number of months following diagnosis of RCC to the earlier of death or the date of the end of the study. The patients in this database who received nephrectomy had their surgery within 4–6 months of diagnosis. The variables were operationalized as categorical variables except for “year of diagnosis” and “survival” variables that were operationalized as continuous variables.

### Outcomes

The unit of analysis was the patient. The primary outcome was the receipt of nephrectomy, which was either radical or partial nephrectomy. CSM (cancer-specific mortality) and ACM (all-cause mortality) were the secondary outcome variables operationalized as time-to-event variables.

### Analysis

Chi-squared and T-test univariate analyses were used to compare baseline characteristics of black patients vs white patients in the study. The association between race and receipt of nephrectomy was evaluated by fitting a multivariable logistic regression model, with demographic and tumor characteristics as covariates. Cox proportional hazard models were fitted to determine the influence of race on CSM and ACM while controlling for confounders. Additionally, Cox proportional hazard models were fitted to assess the relationship between nephrectomy and survival in the overall study population and within each race category. All analyses were conducted using SAS 9.4 software (Cary, NC). The level of statistical significance was set at *p<* 0.05.

## Results

**Fig 1:**
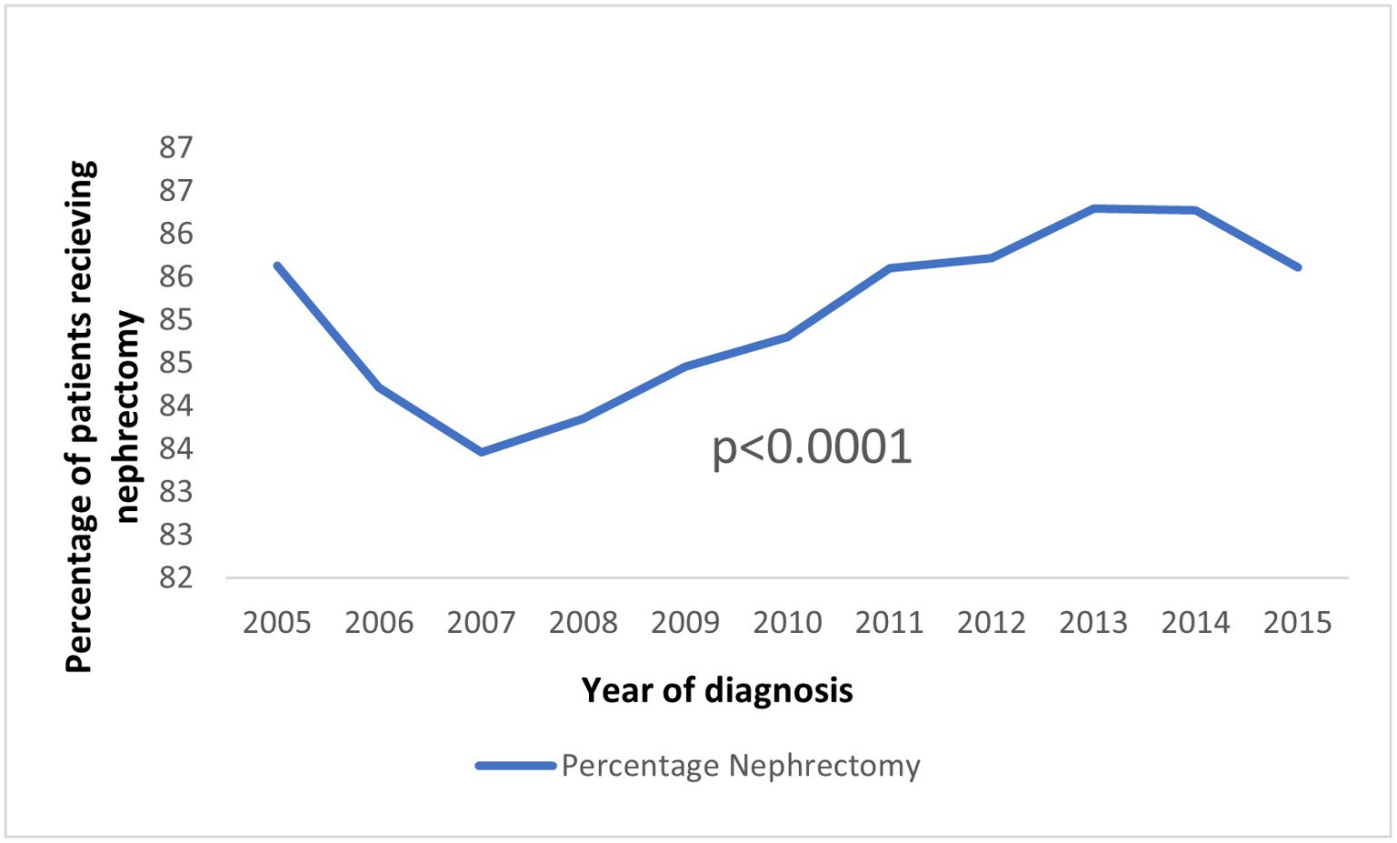
Trend of the use of nephrectomy.

Black patients and white patients constituted 11.51% (n = 8,066) and 88.49% (n = 61,993) of the study cohort respectively. The post-diagnosis follow-up of the cohort median (interquartile range) was 54 (63) months; the median (interquartile range) post-diagnosis follow-up for black patients and white patients was 51 (60) months and 54 (63) months, respectively. The mean age at diagnosis (SD) was 61.4 (12.4) years; the mean ages (SD) for black patients and white patients were 59.5 (11.7) years and 61.7 (12.5) years, respectively. Fig 1 summarizes the trend of the use of nephrectomy.

Table 1 details the comparison of demographic and tumor characteristics of all study subjects, and black patients versus white patients. Approximately 57.8% (n = 40,484) were ≤ 65 years, 74.4% (n = 52,151) had T1 disease. There were significant differences across tumor stages and geographical location of patients by race (p < 0.0001). A lower proportion of black patients received nephrectomy compared to white patients (82.8% vs 85.4%; p < 0.0001). A greater proportion of white patients vs black patients died of RCC (11.7% vs 9.1%, p < 0.0001) but no difference in all cause death between white patients vs black patients (25.8% vs 26.3%, p = 0.34).

**Table 1:** Characteristic of study cohort and comparison by patients’ race.

Covariates	Total (70059: 100%)	Black patients 8066 (11.51%)	White patients 61993 (88.49%)	*p* value
*year of diagnosis*				
2005	4809 (6.86)	466 (5.78)	4343 (7.01)	<.0001
2006	5667 (8.09)	543 (6.73)	5124 (8.27)	
2007	6095 (8.70)	679 (8.42)	5416 (8.74)	
2008	6582 (9.39)	777 (9.63)	5805 (9.36)	
2009	6903 (9.85)	803 (9.96)	6100 (9.84)	
2010	6294 (8.98)	734 (9.10)	5560 (8.97)	
2011	6355 (9.07)	788 (9.77)	5567 (8.98)	
2012	6499 (9.28)	777 (9.63)	5722 (9.23)	
2013	6556 (9.36)	802 (9.94)	5754 (9.28)	
2014	6962 (9.94)	827 (10.25)	6135 (9.90)	
2015	7337 (10.47)	870 (10.79)	6467 (10.43)	
*Age at diagnosis*, *years*				
<45	6565 (9.37)	831 (10.30)	5734 (9.25)	<.0001
45–54	13168 (18.80)	1760 (21.82)	11408 (18.40)	
55–64	20751 (29.62)	2656 (32.93)	18095 (29.19)	
65–74	18946 (27.04)	2033 (25.20)	16913 (27.28)	
75–84	9324 (13.31)	726 (9.00)	8598 (13.87)	
≥85	1305 (1.86)	60 (0.74)	1245 (2.01)	
*Regions*				
Midwest	8470 (12.09)	1219 (15.11)	7251 (11.70)	<.0001
Northeast	10009 (14.29)	1077 (13.35)	8932 (14.41)	
South	18354 (26.20)	3519 (43.63)	14835 (23.93)	
West	33226 (47.43)	2251 (27.91)	30975 (49.97)	
*Tumor stage*				
T1	52151 (74.44)	6598 (81.80)	45553 (73.48)	<.0001
T2	298 (0.43)	53 (0.66)	245 (0.40)	
T3	16451 (23.48)	1303 (16.15)	15148 (24.44)	
T4	1159 (1.65)	112 (1.39)	1047 (1.69)	
*Tumor grade*				
1	8996 (12.84)	1022 (12.67)	7974 (12.86)	<.0001
2	36588 (52.22)	4175 (51.76)	32413 (52.28)	
3	19554 (27.91)	2406 (29.83)	17148 (27.66)	
4	4921 (7.02)	463 (5.74)	4458 (7.19)	
*Nephrectomy*				
No	10439 (14.90)	1384 (17.16)	9055 (14.61)	<.0001
Yes	59620 (85.10)	6682 (82.84)	52938 (85.39)	
Partial *Nephrectomy*				
No	46230 (65.99)	5272 (65.36)	40958 (66.07)	0.2068
Yes	23829 (34.01)	2794 (34.64)	21035 (33.93)	
Radical Nephrectomy				
No	34268 (48.91)	4178 (51.80)	30090 (48.54)	<.0001
Yes	35791 (51.09)	3888 (48.20)	31903 (51.46)	
*Cancer-specific mortality*				
Alive	62047 (88.56)	7331 (90.89)	54716 (88.26)	<.0001
Dead	8012 (11.44)	735 (9.11)	7277 (11.74)	
*All-cause mortality*				
Alive	51946 (74.15)	5945 (73.70)	46001 (74.20)	0.3355
Dead	18113 (25.85)	2121 (26.30)	15992 (25.80)	

Table 2 summarizes the comparison of demographic and tumor characteristics of all study subjects by their nephrectomy status. Approximately 85.1% (n = 59,620) of the patients received nephrectomy. Table 2 presents differences in nephrectomy by age, sex, race, region, and tumor stage. Patients younger than 65 years were more likely to receive nephrectomy than older patients diagnosed with RCC (< 0.0001), and patients with lower-stage disease were more likely to receive nephrectomy (p < 0.0001).

**Table 2:** Characteristics of the study cohort and comparison by receipt of nephrectomy.

Covariates	Total (70059; 100%)	Yes (59620; 85.10%)	No (9541; 13.62%)	*p* value
*Year of diagnosis*				
2005	4809 (6.86)	4118 (6.91)	691 (6.62)	0.0001
2006	5667 (8.09)	4772 (8.00)	895 (8.57)	
2007	6095 (8.70)	5087 (8.53)	1008 (9.66)	
2008	6582 (9.39)	5519 (9.26)	1063 (10.18)	
2009	6903 (9.85)	5830 (9.78)	1073 (10.28)	
2010	6294 (8.98)	5337 (8.95)	957 (9.17)	
2011	6355 (9.07)	5440 (9.12)	915 (8.77)	
2012	6499 (9.28)	5571 (9.34)	928 (8.89)	
2013	6556 (9.36)	5658 (9.49)	(8.60)	
2014	6962 (9.94)	6007 (10.08)	955 (9.15)	
2015	7337 (10.47)	6281–10.54	1056 (10.12)	
*Age at diagnosis*, *years*				
<45	6565 (9.37)	5852 (9.82)	713 (6.83)	0.0001
45–54	13168 (18.80)	11525 (19.33)	1643 (15.74)	
55–64	20751 (29.62)	17941 (30.09)	2810 (26.92)	
65–74	18946 (27.04)	16016 (26.86)	2930 (28.07)	
75–84	9324 (13.31)	7375 (12.37)	1949 (18.67)	
≥85	1305 (1.86)	911 (1.53)	394 (3.77)	
*Regions*				
Midwest	8470 (12.09)	6505 (10.91)	1965 (18.82)	0.0001
Northeast	10009 (14.29)	8440 (14.16)	1569 (15.03)	
South	18354 (26.20)	15795 (26.49)	2559 (24.51)	
West	33226 (47.43)	28880 (48.44)	4346 (41.63)	
*Tumor stage*				
T1	52151 (74.44)	44406 (74.48)	7745 (74.19)	0.0001
T2	298 (0.43)	138 (0.23)	160 (1.53)	
T3	16451 (23.48)	14396 (24.15)	2055 (19.69)	
T4	1159 (1.65)	680 (1.14)	479 (4.59)	
*Tumor grade*				
1	8996 (12.84)	7249 (12.16)	1747 (16.74)	0.0001
2	36588 (52.22)	31324 (52.54)	5264 (50.43)	
3	19554 (27.91)	16848 (28.26)	2706 (25.92)	
4	4921 (7.02)	4199 (7.04)	722 (6.92)	
*Cancer-specific mortality*				
Alive	62047 (88.56)	53276 (89.36)	8771 (84.02)	0.0001
Dead	8012 (11.44)	6344 (10.64)	1668 (15.98)	
*All-cause mortality*				
Alive	51946 (74.15)	45238 (75.88)	6708 (64.26)	0.0001
Dead	18113 (25.85)	14382 (24.12)	3731 (35.74)	
*Race*				
Black patients	8066 (11.51)	6682 (11.21)	1384 (13.26)	0.0001
White patients	61993 (88.49)	52938 (88.79)	9055 (86.74)	

Table 3 displays the results of the multivariable logistic regression. The odds of receipt of nephrectomy reduced with increasing age. Compared to white patients, black patients had 18% lower adjusted odds of receipt of nephrectomy (Crude = 17% lower odds, p<0.0001). Compared to patients with T1, patients with stages T2, T3, T4 disease were 83% less likely, 17% more likely, and 78% less likely to receive nephrectomy, respectively.

**Table 3:** Multivariable logistic regression model showing the odds of receipt of nephrectomy.

Covariates	HR (95% CI)	*p* value
*Year of diagnosis*	1.01 (1.01–1.02)	0.0006
*Age at diagnosis*, *years*		
<45		
45–54	0.86 (0.78–0.95)	0.0017
55–64	0.79 (0.71–0.85)	<.0001
65–74	0.65 (0.60–0.71)	<.0001
75–84	0.46 (0.41–0.50)	<.0001
≥85	0.27 (0.24–0.31)	<.0001
*Race*		
White patients (reference)		
Black patients	0.82 (0.76–0.87)	<.0001
*Region*		
Midwest (reference)		
Northeast	1.68 (1.56–1.81)	<.0001
South	1.87 (1.75–2.00)	<.0001
West	2.03 (1.94–2.16)	<.0001
*Tumor stage*		
T1 (reference)		
T2	0.17 (0.13–0.21)	<.0001
T3	1.17 (1.11–1.24)	<.0001
T4	0.22 (0.19–0.25)	<.0001
*Tumor grade*		
1 (reference)		
2	1.41 (1.33–1.50)	<.0001
3	1.55 (1.44–1.66)	<.0001
4	1.60 (1.43–1.77)	<.0001

Table 4 summarizes the mortality risks of the study patients. The risk of CSM is similar between black patients and white patients. However, CSM increased with older age and higher tumor stage. Patients who did not receive nephrectomy had a 42% higher risk of CSM relative to patients who received nephrectomy. The risk of ACM in black patients was 27% higher compared to white patients. ACM increased with older age and higher stage. Patients who did not receive nephrectomy had a 35% higher risk of ACM relative to patients who received nephrectomy.

**Table 4:** Multivariable Cox proportional hazards models showing the risk of mortality in the study cohort.

Covariates	Cancer-specific mortality		All-cause mortality	
	HR (95% CI)	*p* value	HR (95% CI)	*p* value
*Nephrectomy*				
Yes	0.58 (0.55–0.62)	<.0001	0.65 (0.63–0.68)	<.0001
*Year of diagnosis*	0.96 (0.95–0.97)	<.0001	0.97 (0.97–0.98)	<.0001
*Age at diagnosis*, *years*				
<45 (reference)				
45–54	1.27 (1.14–1.43)	<.0001	1.57 (1.43–1.71)	<.0001
55–64	1.37 (1.23–1.52)	<.0001	2.05 (1.89–2.23)	<.0001
65–74	1.53 (1.37–1.71)	<.0001	2.86 (2.63–3.11)	<.0001
75–84	1.88 (1.68–2.11)	<.0001	4.59 (4.22–4.99)	<.0001
≥85	2.33 (1.99–2.74)	<.0001	7.13 (6.42–7.92)	<.0001
*Race*				
White patients (reference)				
Black patients	1.06 (0.98–1.15)	0.1222	1.27 (1.21–1.33)	<.0001
*Region*				
Midwest (reference)				
Northeast	0.96 (0.88–1.05)	0.3400	0.911 (0.86–0.97)	0.0014
South	1.16 (1.07–1.25)	0.0002	1.14 (1.08–1.20)	<.0001
West	1.11 (1.04–1.19)	0.0036	1.00 (0.95–1.05)	0.9678
*Tumor stage*				
T1 (reference)				
T2	2.73 (1.99–3.75)	<.0001	1.43 (1.19–1.73)	0.0002
T3	6.24 (5.90–6.57)	<.0001	2.47 (2.40–2.55)	<.0001
T4	16.86 (15.44–18.41)	<.0001	6.59 (6.13–7.07)	<.0001
*Tumor grade*				
1 (reference)				
2	1.21 (1.08–1.35)	0.0011	1.01 (0.96–1.07)	0.6641
3	2.80 (2.50–3.13)	<.0001	1.49 (1.41–1.57)	<.0001
4	6.24 (5.55–7.01)	<.0001	3.07 (2.89–3.27)	<.0001

The risk of CSM and ACM of the study patients, by race, showed a similar trend in patients treated with either radical or partial nephrectomy (Tables 5 & 6).

**Table 5:** Multivariable Cox proportional hazards models showing the risk of mortality in the radical nephrectomy study cohort.

Covariates	Cancer-specific mortality		All-cause mortality	
	HR (95% CI)	*p* value	HR (95% CI)	*p* value
*Race*				
White patients (reference)				
Black patients	1.08 (1.00–1.17)	0.0497	1.29 (1.23–1.35)	<.0001

**Table 6:** Multivariable Cox proportional hazards models showing the risk of mortality in the partial nephrectomy study cohort.

Covariates	Cancer-specific mortality		All-cause mortality	
	HR (95% CI)	*p* value	HR (95% CI)	*p* value
*Race*				
White patients (reference)				
Black patients	1.09 (1.01–1.18)	0.0317	1.29 (1.23–1.35)	<.0001

## Discussion

In this study, we investigated the disparity in the receipt of nephrectomy and mortality risk between black patients and white patients, diagnosed with renal cell carcinoma in the US over ten years. Black patients had lower odds of receipt of nephrectomy and a higher risk of all-cause mortality than white patients. In agreement with our findings, a study by Zini et al. suggests that the receipt of nephrectomy, the gold standard treatment for non-metastatic renal cell cancer, differs by race. However, it suggests that this difference did not have any effect on survival outcomes [17]. Another study examining the trends in the use of nephrectomy and cytoreductive surgery in the US reported that black patients were less likely than white patients to receive surgical intervention when diagnosed with RCC [18].

The use of nephrectomy appears to be on the rise, and most patients in this study received some form of nephrectomy. Although younger age at diagnosis was associated with greater odds of receipt of nephrectomy and black patients were more likely to be diagnosed with RCC at a younger age than white patients, black patients still had 18% lesser odds of nephrectomy receipt than white patients in this study. The SEER programs did not collect information on comorbidity or information that may help to calculate comorbidity scores (e.g., Charlson’s Comorbidity Index). Hence the influence of comorbidity on receipt of nephrectomy could not be determined. Previous study examining the influence of age and comorbidity of receipt of radical surgeries for major urological cancers suggest age is more important than comorbidity in the receipt of radical surgeries [19]. While age and comorbidity may not be the only factors limiting RCC health outcomes in black patients, discriminatory policies and practices have created barriers for black patients in accessing and utilizing health care. Also, the inability of the healthcare system to demonstrate trustworthiness is a likely contributor to poorer health outcomes in black patients.

Black patients had higher ACM than white patients when diagnosed with renal cell carcinoma in this study. Increasing age at diagnosis, tumor grade, and stage are associated with greater odds of cancer-specific and all-cause mortality. Because black patients have 18% lesser odds of receipt of nephrectomy than white patients, it is not surprising that black patients have about 27% greater odds of ACM than white patients. Multiple factors such as differential comorbidities and use of other therapies such as adjuvant therapy not captured in this analysis due to the limitation of the data could have affected the ACM outcome. A study that analyzed SEER data concluded that there is reduced receipt of nephrectomy in black patients versus white patients but suggests that the reduction in receipt of nephrectomy does not influence survival outcomes. The study had a smaller population, a shorter follow-up time and due to its timing, was unlikely to have captured the benefits of nephron-sparing surgery [17].

Access to nephrectomy and survival outcomes are lower in black patients than in white patients [17, 20]. Compared to white patients, discriminatory policies have led to lower health insurance coverage, less likelihood of having an established source of healthcare, and greater difficulty in seeking healthcare in black individuals [21–23]. Also, there is a long-standing inability of the healthcare system to show that it is deserving of the trust of black patients [24, 25]. Perhaps, the most discussed example of this is the Tuskegee syphilis experiment, where black patients were intentionally exposed to harm. Similar to this is the experience of African American women unethically subjected to gynecological procedures and such experiences as when slaves were not offered medical care because they were slaves [23]. Similarly, it is reported that providers caring for black patients are less likely to give adequate health information to their patients, thus impairing their ability to participate in shared decision-making about their care [26]. These experiences may have bred a fear of complications, an aversion to surgery [27, 28] and an overall negative attitude towards accepting procedures such as nephrectomy. The discrimination in health care delivery, provider bias plus the negative predisposition towards the health care system contributes to poorer health outcomes in black patients diagnosed with RCC.

### Limitations

We applied a retrospective study design. Due to the nature of the SEER data, it was impossible to capture the use of additional therapies such as adjuvant therapies/ chemotherapies which may have effects on survival outcomes. Lastly, information on comorbidities was not available in our data, hence, we were unable to adjust for comorbidities in our analysis.

In this study, the sample size of some racial groups was too small to make meaningful statistical inference. The inability to evaluate the use of nephrectomy in certain racial groups is a limitation.

Lastly, due to the unavailability of social economic data or social determinants of health (SDOH) in the SEER data in this analysis, we were unable to explore the association between socio-economic status or SDOH and receipt of nephrectomy.

## Conclusion

Although there has been technological advancement and the use of nephrectomy in treating patients diagnosed with RCC has improved, black patients are still less likely than white patients to receive nephrectomy despite the greater likelihood of RCC incidence and mortality in black patients. Several factors stemming from systemic racism and racial inequalities contribute greatly to a disparity in health care delivery. Improving health outcomes for patients diagnosed with RCC will require a deliberate adoption of system-wide social and medical changes in the approach to health care delivery and treatment decisions that address systemic racism and eliminate disparity in health outcomes in black patients and other minority populations in the US.
